# Differential Expression of Urinary Exosomal MicroRNAs miR-21-5p and miR-30b-5p in Individuals with Diabetic Kidney Disease

**DOI:** 10.1038/s41598-019-47504-x

**Published:** 2019-07-29

**Authors:** Jinnan Zang, Alexander P. Maxwell, David A. Simpson, Gareth J. McKay

**Affiliations:** 1 Centre for Public Health, Queen’s University Belfast, Belfast, United Kingdom; 2 Centre for Experimental Medicine, Queen’s University Belfast, Belfast, United Kingdom

**Keywords:** Diagnostic markers, Chronic kidney disease

## Abstract

Biomarkers for the identification of diabetic kidney disease (DKD) are needed as current tests lack sensitivity for detecting early kidney damage. MicroRNAs (miRNAs) are short, non-coding regulatory ribonucleic acid (RNA) molecules commonly found in urinary exosomes differentially expressed as renal function declines. We evaluated urinary exosomal miRNA expression in persons with type 2 diabetes mellitus and DKD (T2DKD). 87 human urinary exosomal miRNAs were profiled in a discovery cohort of patients with T2DKD (n = 14) and age and gender matched controls with type 2 diabetes mellitus and normal renal function (T2DNRF; n = 15). Independent validation of differentially expressed target miRNAs was performed in a second cohort with T2DKD (n = 22) and two control groups: T2DNRF (n = 15) and controls with chronic kidney disease (CCKD) and poor renal function without diabetes (n = 18). In the discovery cohort, urinary miR-21-5p, let-7e-5p and miR-23b-3p were significantly upregulated in T2DKD compared to T2DNRF (p < 0.05). Conversely, miR-30b-5p and miR-125b-5p expression was significantly lower in T2DKD (p < 0.05). Independent validation confirmed up-regulation of miR-21-5p in the replication cohort in T2DKD (2.13-fold, p = 0.006) and in CCKD (1.73-fold, p = 0.024). In contrast, miR-30b-5p was downregulated in T2DKD (0.82-fold, p = 0.006) and in CCKD (0.66-fold, p < 0.002). This study identified differential expression of miR-21-5p and miR-30b-5p in individuals with diabetic kidney disease and poor renal function. These miRNAs represent potential biomarkers associated with the pathogenesis of renal dysfunction.

## Introduction

Chronic kidney disease (CKD) is defined clinically as abnormalities of kidney function or structure present for more than 3 months, with potential implications for health^1^. This definition includes individuals with markers of kidney damage, such as proteinuria, and those with an estimated glomerular filtration rate (eGFR) of less than 60 ml/min/1.73 m^2^ recorded on at least two occasions in the previous 3 months.

CKD is commonly associated with hypertension and diabetes and it is estimated that almost three million people are living with CKD in the United Kingdom alone^2^. Diabetic kidney disease (DKD), also called diabetic nephropathy, refers to CKD that is present in persons with diabetes and is defined as macroalbuminuria (urinary albumin-to-creatinine ratio [ACR] >30 mg/mmol), or microalbuminuria (ACR: 3–30 mg/mmol) associated with retinopathy and/or more than 10 years duration of type 1 diabetes mellitus^3^. Renal impairment develops in approximately one-third of those with diabetes and is associated with increased comorbidity and premature mortality^4^.

Characteristic pathological changes associated with diabetes in the kidney include podocyte loss, glomerular basement membrane thickening, mesangial matrix expansion, arterial hyalinosis, diffuse glomerulosclerosis and nodular Kimmelstiel-Wilson lesions^5^. The earliest stages of DKD are asymptomatic and may go unrecognised without regular screening for albuminuria and/or declining eGFR. DKD may progress to end stage renal disease (ESRD) necessitating renal replacement therapy (chronic dialysis or kidney transplantation) which represents a significant clinical and financial burden^6^. Over the past decade, global mortality rates associated with ESRD have increased by 32%^7^ and DKD associated with type 2 diabetes mellitus (T2DM) has been shown to account for 35–50% of ESRD in the United States^8^. This highlights the urgent need for improved assays for early detection of DKD to enable prompt treatment.

Serum creatinine-based eGFR measurement and urinary ACR represent current best practice for assessing glomerular damage and evaluation of renal function. However, non-albuminuric renal impairment is not uncommon, especially in those with T2DM. In addition, the precision of creatinine-based GFR estimates is limited in those at extremes of body mass index and in individuals with glomerular hyperfiltration in the early stages of DKD^9^. As such, albuminuria and eGFR are less reliable indicators in the early-stages of DKD highlighting the necessity for the discovery of novel biomarkers for the early detection of DKD.

MicroRNAs (miRNAs) are short non-coding ribonucleic acid (RNA) molecules that are approximately 22 nucleotides in length and modulate gene expression through targeted degradation of messenger ribonucleic acid (mRNA) molecules or suppression of their translation^10^. Several miRNAs have previously been associated with the development and differentiation of kidney cells and may be markers of kidney cell damage^11–13^. Exosomes are membrane-bound vesicles that are released into body fluids such as serum, plasma, urine and saliva and carry miRNAs which may offer potential as non-invasive biomarkers of disease^14–17^. Urine is an ideal source for non-invasive miRNA profiling because it is produced in the kidneys and its constituents are known to reflect renal function, with proteins such as cystatin C increasing in individuals with renal impairment and kidney damage^18,19^. Urinary miRNAs may therefore provide a simple, non-invasive biomarker of kidney function measurable in an easily accessible liquid biopsy.

The objective of this study was to identify urinary exosomal miRNAs associated with renal dysfunction in T2DKD with independent replication and validation.

## Materials and Methods

### Patient samples

All study participants were recruited from renal clinics at Belfast City Hospital following written informed consent and all research was performed in accordance with relevant guidelines under ethical approval by the Office for Research Ethics Committees Northern Ireland (REC/14/NI/1132). All participants were white and of European ancestry. Participant information was collected upon enrolment including diabetes status, past history of cardiovascular events, current prescribed medications, together with clinical chemistry results including serum creatinine, urinary ACR, lipoproteins, and glycated haemoglobin A1c (HbA1c) measured in persons with diabetes. Spot urine samples were collected in 50 ml sterile centrifuge tubes and placed on ice prior to processing. Urine samples were rendered acellular following centrifugation at 2,000 g for 10 min at 4 °C whereupon the supernatant was transferred to 5 × 2 ml aliquots in RNase/DNase-free tubes and frozen at −80 °C.

Renal function was estimated using the Chronic Kidney Disease Epidemiology Collaboration (CKD-EPI) equation which provides improved accuracy over the earlier Modification of Diet in Renal Disease (MDRD) equation^20^, particularly for eGFR values > 60 ml/min/1.73 m^2^ ^21^. CKD was categorised on the basis of multiple serum creatinine measurements using a renal function cut-off value of 60 ml/min/1.73 m^2^ for 3 months or more in the absence of persistent albuminuria, or any eGFR in the presence of persistent albuminuria of ≥3 mg/mmol in line with the clinical definition of CKD^1^. T2DM was diagnosed according to the American Diabetes Association 2010 criteria^22^. Participants with current infection or history of infection in the previous month were not recruited. Participants with known inflammatory diseases e.g. rheumatoid arthritis, systemic lupus erythematosus, Crohn’s disease were excluded from the study. Sample size was determined using GPower^23^ on the basis of a false-positive rate of 0.05 and a power of 90%, with effect size of 4 indicating a need for 12 participants per group.

Participants in the discovery cohort were categorised into the following groups (i) T2DKD (T2DM and eGFR < 60 ml/min/1.73 m^2^), (ii) age and gender matched T2DNRF (T2DM and eGFR > 60 ml/min/1.73 m^2^). The validation cohort comprised three groups: (i) T2DKD, (ii) T2DNRF, (iii) CCKD (no diabetes and CKD).

### Isolation of urinary exosomes and total RNA

Exosomes were isolated from 1.1 ml of urine supernatant thawed on ice and centrifuged at 10,000 *g* (Eppendorf, Hamburg, Germany) at room temperature to remove cellular debris in accordance with the protocol for the miRCURY™ Exosome Isolation Kit (Qiagen, Hilden, Germany). Total RNA was extracted from the exosomes following the protocol of the Cell and Plant miRCURY™ RNA Isolation Kit (Qiagen). RNA quality and concentration were determined using NanoDrop One (ThermoFisher Scientific, San Jose, CA).

### miRNA profiling using polymerase chain reaction (PCR) panels in the discovery cohort

Reverse transcription of RNA to complementary deoxyribonucleic acid (cDNA) was performed according to the miRCURY Locked Nucleic Acids (LNA) Universal RT kit (Qiagen). An estimated 20 ng of total RNA is recommended; reagents included 2 µl of 5X Reaction Buffer, 3.5 µl of nuclease-free water, 1 µl of Reverse Transcriptase Enzyme Mix, 0.5 µl of synthetic RNA spike-in control (UniSp6) and 3 µl of RNA template. Reactions were performed at 42 °C for 60 minutes (min) and then 95 °C for 5 min. Ten µl of cDNA was diluted 1:100 and added to 1000 µl of ExiLENT SYBR Green Master Mix (Qiagen), to give a final reaction volume of 10 µl in each of the 384 wells, which included four replicate primer sets of 87 miRNAs (miRCURY LNA miRNA urine focus PCR Panel (Qiagen)). Quality control inter-plate calibrator UniSp3 and spike-in UniSp6 primers were also included. Amplification was performed on a LightCycler 480 Real-Time PCR system (Roche Diagnostics GmbH, Mannheim, Germany) under the following conditions: 95 °C for 10 min, 45 cycles of 95 °C for 10 seconds (s) and 60 °C for 1 min, with melt curve analysis using Roche LC software (Roche Diagnostics GmbH). Each sample was replicated for each miRNA. Raw quantification cycles (Cq) were obtained for further analysis.

### miRNA profiling using individual PCR assays in the validation cohort

Significant differentially expressed miRNAs from the Qiagen discovery panel with at least a 1.5 fold expression change, were independently validated. Individual miRNA-specific oligonucleotide primers were purchased (miR-21-5p, let-7e-5p, miR-23b-3p, miR-30b-5p and miR-125b-5p) together with three reference miRNA primer sets (miR-200b-3p, miR-30c-5p and miR-27b-3p) (Qiagen). Diluted cDNA samples (1.2 µl from a 1:40 dilution), 1.5 µl of PCR ExiLENT SYBR Green Master Mix (Qiagen) and 0.3 µl of LNA PCR primer set (Qiagen) were added to 384 well PCR plates using an Echo 525 liquid handling system (Labcyte, San Jose, USA). All PCR reactions were duplicated and performed in a total volume of 3 µl. PCR amplification conditions and analyses were as described for the discovery cohort.

### Pre-processing of PCR data

Cq values were imported to GenEx PCR analysis software (MultiD Analyses AB, Goteborg, Sweden) for pre-processing. The workflow included normalisation of Cq values using inter-plate calibration (UniSp3) and those miRNAs most stably expressed between groups as determined by the NormFinder software (GenEx, MultiD Analyses). The miRNA Cq values (Cq [miRNA]) were normalised (ΔCq) using the following formula: ΔCq = average Cq (miRNA) − average Cq (reference miRNAs). Results were represented as an expression fold change (FC) of T2DKD compared to T2DM control samples or alternatively CCKD according to the 2^−ΔΔCq^ method.

### Bioinformatic analyses of differently expressed miRNAs

Identification of differently expressed miRNAs’ target genes was performed using TargetScan7.1 (http://www.targetscan.org/vert_72)^24^. Gene set enrichment for Kyoto Encyclopedia of Genes and Genomes (KEGG) pathways, Gene Ontology and other annotations were performed using the Database for Annotation, Visualization and Integrated Discovery (DAVID) tool (https://david.ncifcrf.gov)^25^. A false discovery rate (FDR) corrected p value < 0.05, was considered statistically significant.

### Statistical analysis

The normality of distributions of all key variables were assessed. Students’ t-test and one-way analysis of variance were used for continuous values with normal distribution. Categorical values were assessed using a Chi-square test. Correlation coefficients (r) were estimated using Spearman’s rank tests and Pearson’s correlation analysis. In addition, receiver operating characteristics (ROC) were used to assess the specificity and sensitivity of the regression models with inclusion of the associated miRNAs. All tests were performed using SPSS version 22.0 (SPSSInc, Chicago, IL, USA). A p value < 0.05 was considered significant. Correction for multiple testing was undertaken according to the Benjamini-Hochberg approach^26^.

## Results

### Study cohort characteristics

Summary statistics of the sample characteristics in the discovery and validation cohorts are provided in Table 1. With the exception of renal function, diastolic blood pressure (DBP) and prescription of insulin and diuretics, there were no significant differences between cases and controls in the discovery cohort for age, gender, systolic blood pressure (SBP), low-density lipoprotein cholesterol (LDL-C), high-density lipoprotein cholesterol (HDL-C), glycosylated haemoglobin (HbA1c) and total cholesterol. Likewise, there were no significant differences in gender, SBP, DBP, LDL-C, HDL-C, HbA1c and total cholesterol in the validation cohort. The mean age of T2DKD (72 years) in the validation cohort was significantly higher than participants in both the T2DNRF (63.3 years) and the CCKD (60.1 years). The mean eGFR was significantly lower in the T2DKD group (p < 0.001; 26.7 ml/min/1.73 m^2^) than the T2DNRF (87.5 ml/min/1.73 m^2^) and CCKD (35 ml/min/1.73 m^2^). There was a difference in the distribution of ACR between groups, with 70% of those in the discovery cohort having an ACR > 30 mg/mmol compared to 60% in the validation cohort. In the discovery cohort, 70% (10/14) of those with T2DKD had an ACR > 30 mg/mmol compared to 64% in the validation cohort (13/22).

**Table 1:** Study cohort characteristics.

Variable	Discovery			Validation			
	T2DKD (n = 14)	T2DNRF (n = 15)	p value	T2DKD (n = 22)	T2DNRF (n = 15)	CCKD (n = 18)	p value
Age (years)	66.7 ± 9.6	67.3 ± 10.2	0.867	72.0 ± 9.0	63.3 ± 5.7	60.1 ± 17.0	0.006
Gender (male)	9	10	0.893	12	12	9	0.172
SBP (mmHg)	138 ± 19	128 ± 18	0.157	141 ± 21	134 ± 26	139 ± 21	0.639
DBP (mmHg)	67 ± 7	78 ± 9	0.002	76 ± 11	83 ± 15	76 ± 9	0.124
LDL-C (mmol/l)	2.05 ± 0.96	2.06 ± 0.80	0.989	1.81 ± 0.74	1.69 ± 0.61	2.49 ± 1.50	0.083
HDL-C (mmol/l)	1.12 ± 0.30	1.26 ± 0.31	0.538	1.21 ± 0.42	1.20 ± 0.27	1.45 ± 0.36	0.126
Total cholesterol (mmol/l)	4.28 ± 1.33	3.63 ± 1.22	0.201	3.98 ± 0.81	3.12 ± 0.70	4.83 ± 1.80	0.001
HbA1c (mmol/mol)	70.5 ± 15.8	62.8 ± 20.0	0.259	57.4 ± 15.8	58.4 ± 15.9	39.0 ± 4.6	0.142
Serum creatinine (µmol/l)	196 ± 69	77 ± 138	<0.001	198.6 ± 43.6	77.9 ± 14.9	185.7 ± 103.4	<0.001
eGFR (ml/min/1.73 m^2^)	31 ± 14	82 ± 14	<0.001	26.7 ± 8.4	87.5 ± 15.0	34.6 ± 12.5	<0.001
**ACR (mg/mmol)**							
<3	0	8	<0.001	5	14	4	<0.001
3–30	4	7		3	1	7	
>30	10	0		14	0	7	
Smoking Status							
Never (n)	7	9	0.812	15	3	16	0.001
Ex-smoker (n)	4	4		2	6	0	
Current smoker (n)	3	2		5	6	2	
**Medications**							
Insulin (n)	9	0	<0.001	3	0	0	0.093
ACE inhibitors / ARBs (n)	5	4	0.599	12	6	10	0.611
Beta-blockers (n)	8	8	0.573	9	12	13	0.030
Calcium channel blockers (n)	5	6	0.812	8	5	10	0.348
Statins (n)	11	10	0.474	19	12	12	0.318
Diuretics (n)	10	4	0.016	13	4	5	0.062
Hypoglycaemic agents (n)	7	9	0.610	2	9	0	<0.001

T2DKD: type 2 diabetic kidney disease; T2DNRF: type 2 diabetes normal renal function. CCKD: control chronic kidney disease. Data are shown as mean ± standard deviation (SD). SBP: systolic blood pressure; DBP: diastolic blood pressure; LDL-C: low-density lipoprotein cholesterol; HDL-C: high-density lipoprotein cholesterol; HbA1c: glycosylated haemoglobin; eGFR: estimated glomerular filtration rate; ACR: urinary albumin-to-creatinine ratios; ACE: angiotensin-converting-enzyme; ARBs: angiotensin receptor blockers. Students’ t-test and one-way analysis of variance were used for continuous values with normal distribution. Categorical values were assessed using a Chi-square test.

### Discovery cohort identification of differentially expressed urinary exosomal miRNAs in participants with renal dysfunction

**Figure 1:**
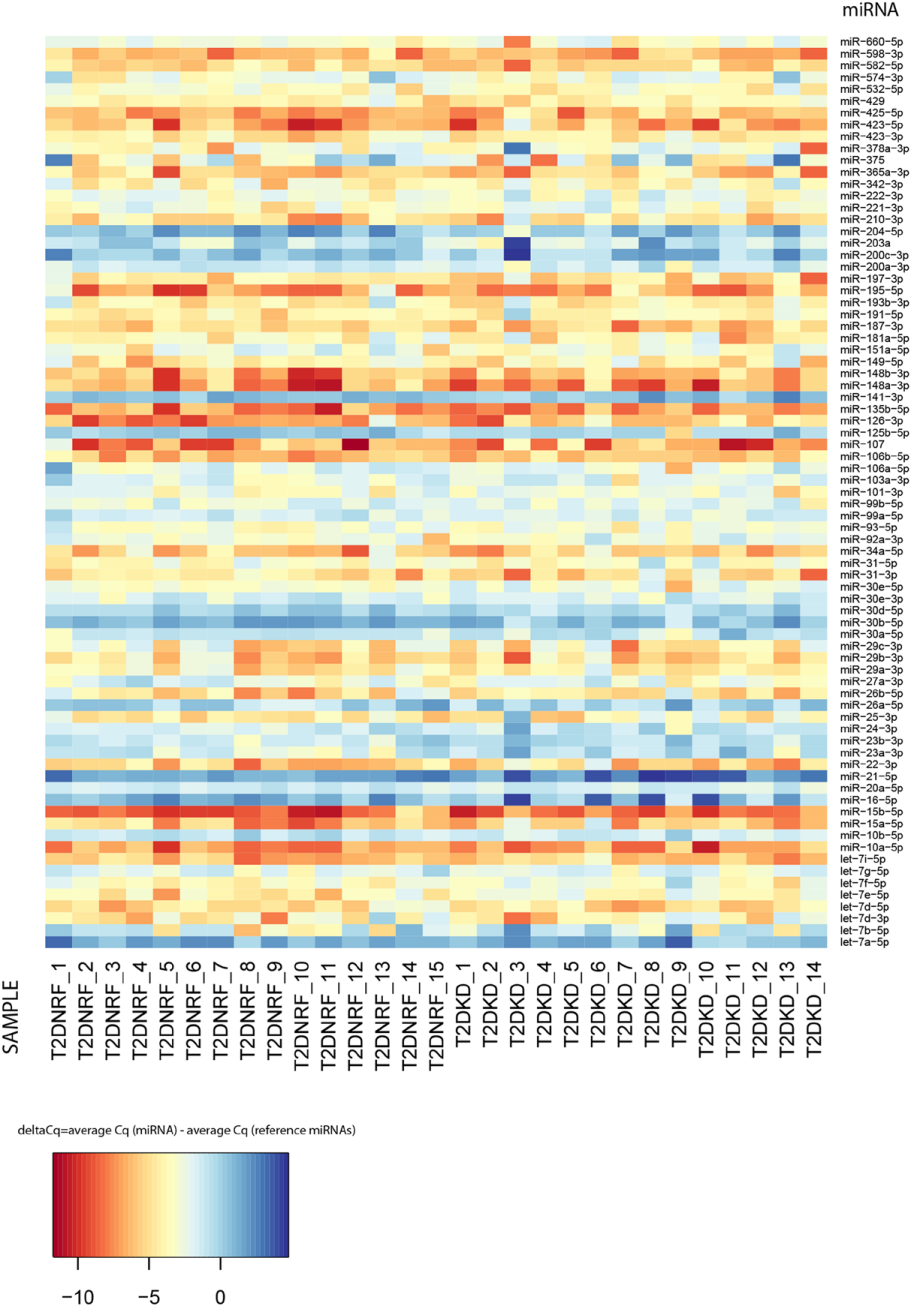
Heat map of discovery cohort miRNA expression profiles.

**Figure 2:**
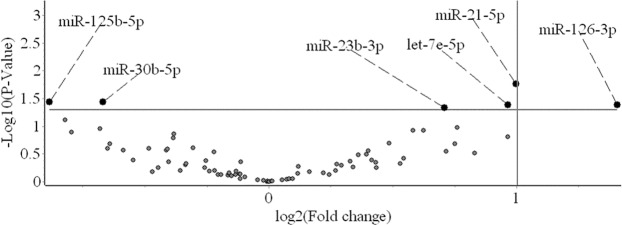
A volcano plot representing differential miRNA expression between type 2 diabetic kidney disease (T2DKD) and type 2 diabetes normal renal function (T2DNRF). The annotated miRNAs were significantly dysregulated in T2DKD. miR-126-3p was not selected for further validation due to the significant deviation in its expression profile (FC: 2.65, 95% CI: 1.04–6.74, p = 0.042).

**Figure 3:**
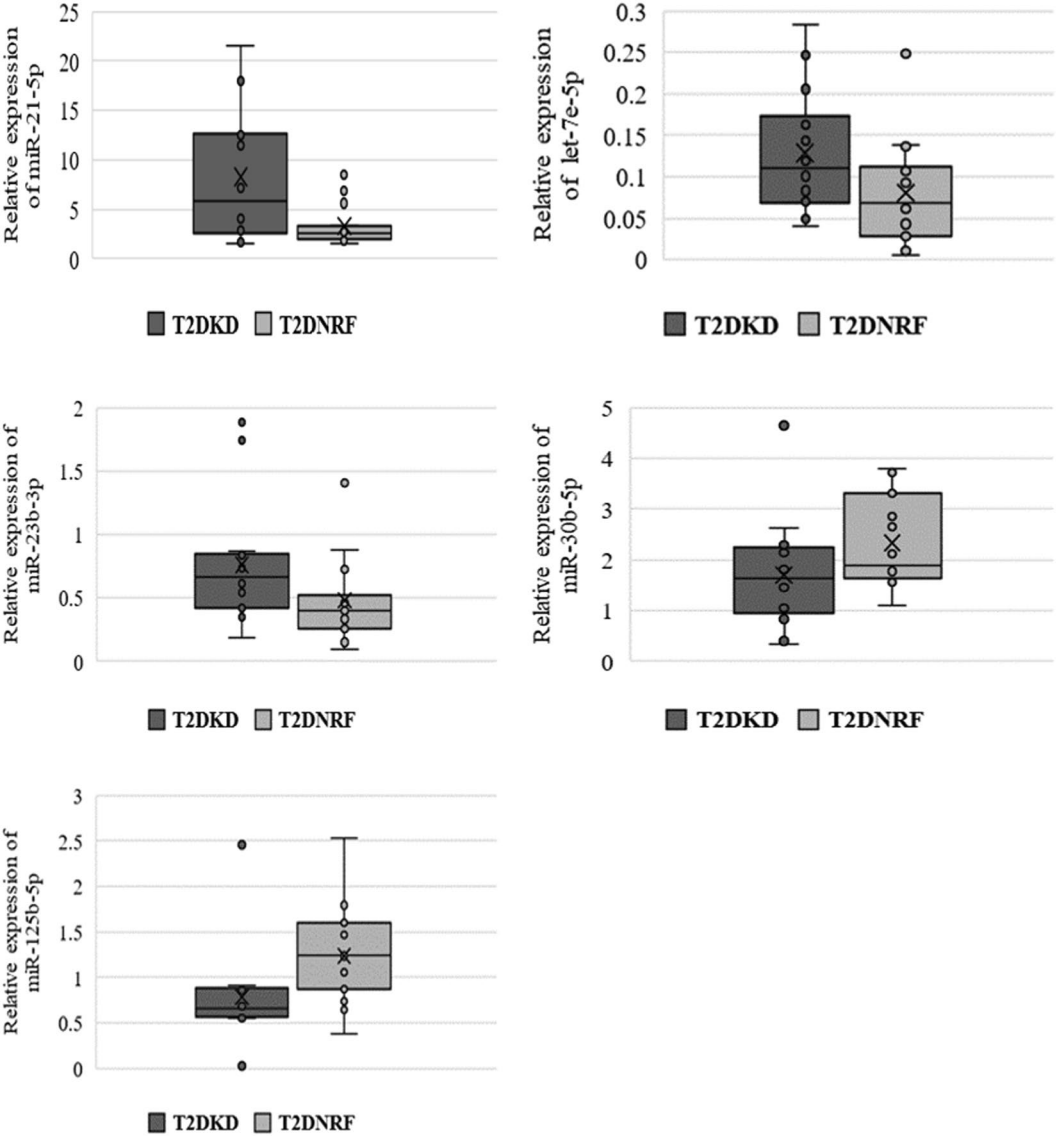
Relative expression in the discovery cohort of five miRNAs significantly dysregulated between type 2 diabetic kidney disease (T2DKD) and type 2 diabetes normal renal function (T2DNRF).

A heatmap of the relative expression values for the 87 miRNAs in the discovery cohort is shown (Fig. 1). Of the 87 miRNAs assayed in the discovery cohort, 77 produced consistent measurements with Cq values < 35 in more than 60% of samples. Expression profiles between T2DKD and age and gender matched T2DNRF were compared. The three miRNAs most stably expressed between groups were selected as candidate reference genes for normalisation purposes (miR-200b-3p, miR-30c-5p and miR-27b-3p). The miRNA expression profiles are represented as a volcano plot (Fig. 2). T2DKD patients had higher levels of miR-21-5p (FC: 2.00, 95% confidence interval [CI]: 1.13-3.53, p = 0.018), let-7e-5p (FC: 1.95, 95% CI: 1.04–3.65, p = 0.041) and miR-23b-3p (FC: 1.63, 95% CI: 1.01–2.64, p = 0.047) compared to the control group and decreased levels of miR-30b-5p (FC: 0.63, 95% CI: 0.40–0.98, p = 0.037), and miR-125b-5p (FC: 0.54, 95% CI: 0.30–0.97, p = 0.037). Of note, P values were no longer significant following correction for multiple testing. Although the expression of miR-126-3p was 2.65-fold higher in T2DKD compared to the discovery control group, the signal intensities were low and not detected in several samples leading to broad confidence intervals. As such, we did not include miR-126-3p for further validation. Variation in the relative expression profiles of these miRNAs between T2DKD and T2DNRF in the discovery cohort are presented (Fig. 3).

### Validation of differentially expressed urinary exosomal miRNAs

**Figure 4:**
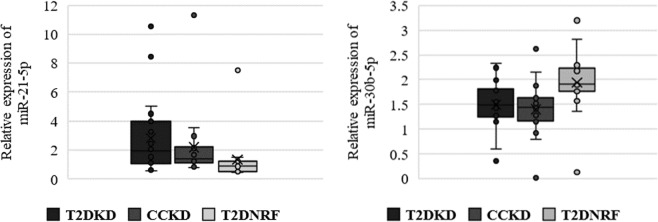
Normalised relative expression of both validated miRNAs (miR-21-5p and miR-30b-5p) in type 2 diabetic kidney disease (T2DKD); type 2 diabetes normal renal function (T2DNRF) and control chronic kidney disease (CCKD).

Similar to the discovery cohort, the three most stably expressed miRNAs were miR-200b-3p, miR-30c-5p and miR-27b-3p which were used as reference genes for normalisation purposes. The expression profiles in T2DKD relative to T2DNRF in the validation cohort are presented for the miRNAs differentially expressed in the discovery cohort (Fig. 4). Only miR-21-5p (FC: 2.13, 95% CI: 1.29–3.52, p = 0.006) and miR-30b-5p levels (FC: 0.82, 95% CI: 0.54–1.26, p = 0.006) remained significantly differentially expressed between T2DKD and T2DNRF. Both miRNAs were also similarly differentially expressed in comparisons between CCKD and T2DNRF respectively: (miR-21–5p; FC: 1.73, 95% CI: 1.07–2.79, p = 0.024 and miR-30b-5p; FC: 0.66, 95% CI: 0.33–1.26, p = 0.002). There was no significant difference in the expression profile of either miR-21-5p or miR-30b-5p between T2DKD and CCKD. The relative expression profiles across all three groups of both significantly differentially expressed miRNAs are shown in Fig. 4.

### Correlations between serum creatinine and miR-21-5p and miR-30b-5p

Correlations were evaluated between miR-21-5p and miR-30b-5p and clinical parameters in all samples of the validation cohort. A significant positive correlation was observed between miR-21-5p and serum creatinine (r^2^ = 0.517; p < 0.001) and a corresponding negative correlation with eGFR (r^2^ = −0.334; p = 0.035; Table 2). Conversely, a negative correlation was observed between miR-30b-5p and serum creatinine (r^2^ = −0.43; p = 0.006) and a positive correlation with eGFR (r^2^ = 0.299; p = 0.06). In addition, miR-30b-5p was found to be significantly correlated with HDL-C (r^2^ = 0.427; p = 0.012). Correlations between let-7e-5p, miR-23b-3p, miR-125b-5p and clinical characteristics in the validation cohort were also considered (Table 2).

**Table 2:** Pearson’s correlations analysis of urinary exosomal miR-21-5p, miR-30b-5p, let-7e-5p, miR-23b-3p and miR-125b-5p expression and clinical parameters in the validation cohort.

Parameter	miR-21-5p		miR-30b-5p		let-7e-5p		miR-23b-3p		miR-125b-5p	
	r	p value	r	p value	r	p value	r	p value	r	p value
Age	0.180	0.267	−0.148	0.362	0.209	0.195	0.196	0.226	−0.110	0.498
Gender^Ɨ^	−0.041	0.801	<0.001	1.000	0.063	0.700	0.017	0.915	0.022	0.894
SBP	−0.013	0.936	−0.075	0.652	−0.036	0.827	0.001	0.994	0.187	0.254
DBP	−0.231	0.158	0.202	0.217	−0.275	0.090	−0.316	0.050	0.180	0.274
HDL-C	−0.298	0.087	0.427	0.012	−0.289	0.097	−0.247	0.159	0.059	0.742
LDL-C	0.020	0.913	−0.045	0.802	−0.020	0.910	0.005	0.978	0.132	0.464
Total cholesterol	−0.117	0.504	−0.041	0.813	−0.100	0.570	−0.052	0.768	−0.049	0.782
Serum creatinine	0.517	<0.001	−0.430	0.006	0.392	0.012	0.298	0.062	−0.092	0.574
HbA1c	0.111	0.633	−0.191	0.406	0.030	0.896	0.106	0.647	−0.007	0.978
eGFR	−0.334	0.035	0.299	0.060	−0.224	0.164	−0.164	0.311	0.093	0.566
ACR	0.039	0.811	−0.071	0. 664	0.055	0.738	0.027	0.868	0.391	0.013
CVD^Ɨ^	0.035	0.831	−0.157	0.334	0.096	0.557	0.150	0.355	−0.024	0.883

SBP: systolic blood pressure; DBP: diastolic blood pressure; HDL-C: high-density lipoprotein cholesterol; LDL-C: low-density lipoprotein cholesterol; HbA1c: glycosylated haemoglobin; eGFR: estimated glomerular filtration rate; ACR: urinary albumin-to-creatinine ratios; CVD: cardiovascular disease. ^Ɨ^Spearman’s correlation analysis.

### Evaluation of miR-21-5p and miR-30b-5p as potential biomarkers of renal dysfunction

**Figure 5:**
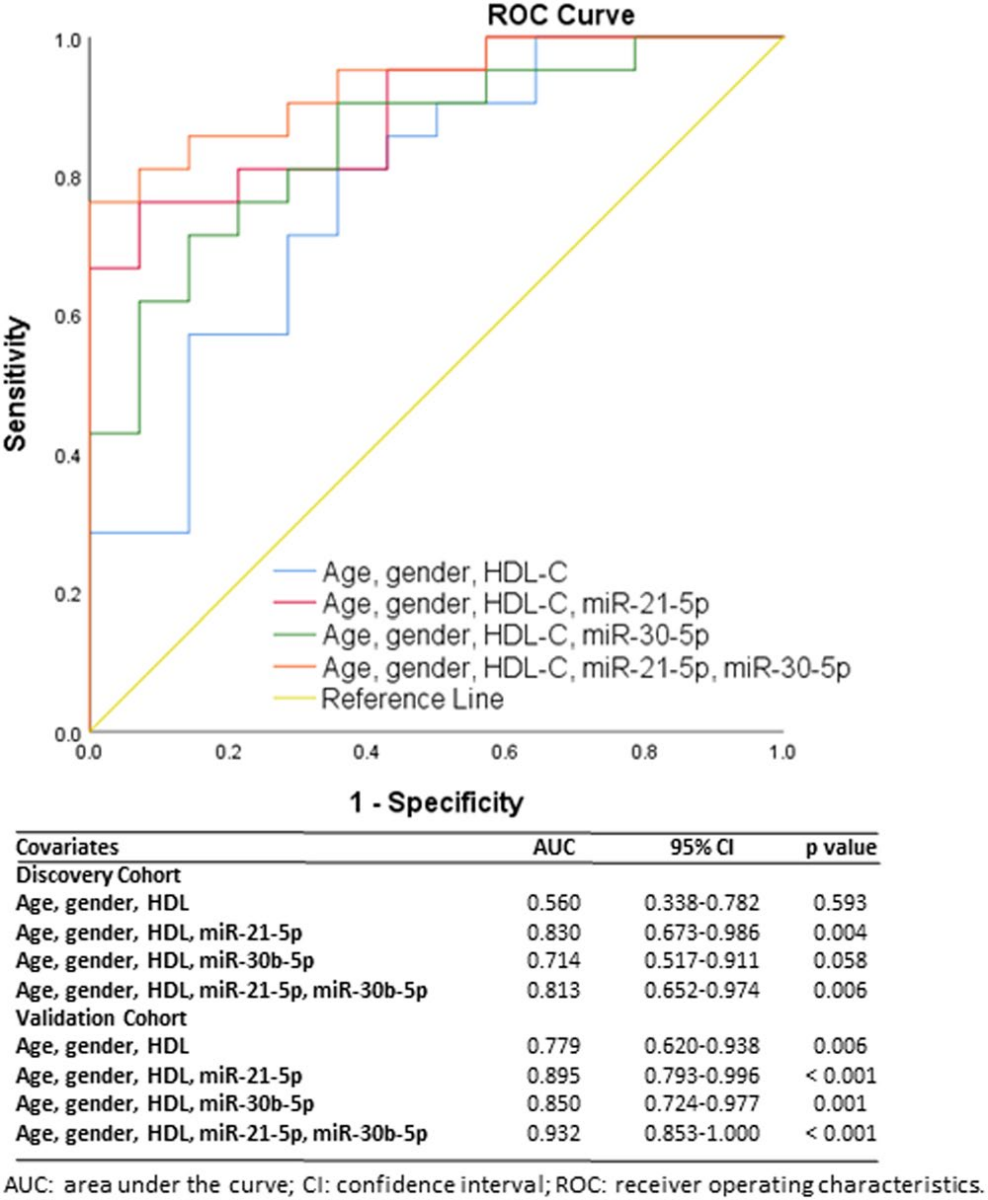
Receiver operating characteristic analysis of miR-21-5p and miR-30b-5p to discriminate between type 2 diabetes normal renal function (T2DNRF) and type 2 diabetic kidney disease (T2DKD) using samples from the validation cohort and type 2 diabetes normal renal function (T2DNRF) and poor renal function (type 2 diabetic kidney disease [T2DKD] and control chronic kidney disease [CCKD] combined) in the validation cohort. Clinical variables comprised age, gender, and high-density lipoprotein cholesterol (HDL-C).

ROC analyses were performed to evaluate the potential of differentially expressed miRNAs to discriminate between individuals with good and poor renal function using samples from the discovery cohort (T2DNRF and T2DKD). When considered with age, gender and HDL-C, miR-21-5p gave an area under the curve (AUC) of 0.830 (CI: 0.673–0.986; P = 0.004) which was reduced when combined with miR-30b-5p (AUC: 0.813; CI: 0.652–0.974; P = 0.006) in the discovery cohort (Fig. 5). Evaluation of miR-21–5p in the validation cohort composed of both T2DKD and CCKD gave an AUC = 0.895 (CI: 0.793–0.996; P < 0.001) which increased further when combined with miR-30b-5p (AUC: 0.932; CI: 0.853–1.000; P < 0.001).

### Bioinformatic analyses of miR-21-5p and miR-30b-5p

TargetScan7.1 (http://www.targetscan.org/vert_72)^24^ was used to predict conserved targets and to increase the specificity of the 102 genes targeted by both miRNAs for further analysis. The most significant term is ‘phosphoprotein’, which is also significantly enriched in the independent targets of both miR-21-5p and miR-30b-5p. The ‘Metabolic’ Class from the Genetic Association Database (GAD) was significantly enriched even after Benjamini-Hochberg correction^26^. Consistent with a role in DKD, the 10^th^ most enriched group within the predicted targets of miR-21 and miR-30 was ‘GAD_DISEASE Type 2 Diabetes/ edema/ rosiglitazone’. All the predicted functional annotations are provided in Supplementary Table S1.

## Discussion

The incidence of DKD is rising worldwide, with associated increased morbidity and premature mortality. Early identification of at risk individuals is crucial for the provision of appropriate and efficient clinical intervention and improved prognosis. Diagnosis of DKD is commonly made using measurements of serum creatinine and urinary albumin excretion. Nevertheless, there is increasing evidence that these biomarkers are of insufficient sensitivity to detect DKD in its early stages^27^ with identification of novel biomarkers, such as miRNAs, considered a priority.

miRNAs are commonly found within extracellular vesicles, and specifically exosomes, that are released from many cell types^28,29^. There is increasing evidence of the potential of both circulating and urinary exosomal miRNAs to act as biomarkers for various renal diseases, including DKD^30^, acute kidney injury^31^, and hypertensive nephropathy^32^, and also provide insights into disease pathogenesis. Given that exosomes within the urine supernatant originate from cells within the various nephron segments^33^, altered miRNA profiles may be indicative of changes in kidney function.

A recent systematic review identified two urinary exosomal miRNAs (miR-342-3p and miR-192-5p) to be differentially regulated in individuals with T2DKD^34^. Of these, only miR-342-3p was represented on the discovery panel in the current study but was not found to be differentially expressed (FC: 1.33, 95% CI: 0.66–2.68, p = 0.408). Sixteen urinary exosomal miRNAs were reported to be differentially regulated in a small cohort of T2DKD compared to healthy controls, although only two miRNAs were validated in subsequent replication (miR-320c and miR-6068)^35^. Of the sixteen miRNAs reported, only miR-30d-5p and miR-30e-5p were present on the Qiagen discovery panel, but there were no significant variation in the levels detected (miR-30d-5p: FC: 0.81, 95% CI: 0.56–1.17, p = 0.25; miR-30e-5p: FC: 1.32, 95% CI: 0.78–2.40, p = 0.27)^35^. In addition, another study has implicated three urinary miRNAs, miR-126-3p, miR-155-5p and miR-29b-3p, as potential biomarkers of DKD^36^. Two of the three miRNAs were present on the discovery panel: miR-126-3p had similar increased expression in the DKD samples (FC: 2.65, 95% CI: 1.04–6.74, p = 0.042), although we chose not to evaluate it in the validation cohort due to the large deviation in its expression profile. We detected no significant variation in expression of miR-29b-3p (FC: 0.87. 95% CI: 0.37–2.08, p = 0.752) in our discovery cohort. The lack of replication of these findings may arise because of clinical heterogeneity of the patients enrolled, specimen collection and experimental methods, in addition to ethnicity, population structure, disease heterogeneity and classification, study power and effect size. As such, interpretation of miRNA expression reported in association with DKD requires caution and careful consideration of potential confounders.

In the validation cohort, an elevated level of miR-21-5p was detected in individuals with poor renal dysfunction, in support of findings reported previously^37–39^. A significant correlation between miR-21-5p and serum creatinine (and eGFR) in the current study strengthens previous associations with renal sclerosis in multiple experimental models and fibrosis in other organs, including heart and lung^40–43^.

Although dysregulation of miR-21-5p is not specific to T2DKD, the results reported in this study and by others, suggest miR-21-5p may offer potential as a marker of declining renal function. A recent study has shown miR-21-5p to be significantly elevated in the renal cortex of insulin-dependent diabetic mice, suppressing phosphatase and tensin homolog (PTEN) protein and increasing fibronectin content, during DKD pathogenesis^44^. In addition, miR-21-5p has been identified as a potential therapeutic target in DKD mouse models, given its pathological role in renal fibrosis^45^. Upregulation of miR-21-5p and a negative correlation with eGFR has been previously reported in both glomeruli and proximal tubules from patients with DKD, focal segmental glomerulosclerosis (FSGS), or membranoproliferative glomerulonephritis (MPGN)^46^. Wang and colleagues demonstrated that overexpression of miR-21-5p could lead to transforming growth factor beta (TGF-β) induced epithelial-mesenchymal transition (EMT) by inhibiting smad7, and that miR-21-5p may provide an alternative target to directly suppress TGF-β-mediated renal fibrosis in DKD^47^. In addition, miR-21-5p has been shown to target tissue inhibitor of metalloproteinases (TIMPs) which are significantly up-regulated in kidneys from diabetic mice and mesangial cells grown under high glucose conditions^48,49^. Furthermore, the same study also showed up-regulation of miR-21-5p in kidney biopsy samples from diabetic patients compared to healthy controls^48^.

While several candidate miRNAs have been previously proposed as potential T2DKD biomarkers, the association with miR-30b-5p is a novel finding. It has been reported as a general miRNA biomarker in several human cancers with reduced expression reported in hepatocellular carcinoma, breast and gastric cancer^50–53^. In addition, urinary miR-30b-5p was shown to be abundant in prostate cancer patients^54^. Members of the miR-30 family have been previously reported at lower levels in T2DKD individuals^35^ and as such may represent markers of podocyte injury and glomerular disease. The miR-30 family may have a protective role in renal podocytes, with reduced expression in individuals with focal segmental glomerulosclerosis^55^. In addition, reduced expression of miR-30 family members has been implicated in epithelial-to-mesenchymal transition which is linked to mechanisms of renal fibrosis^56^. Other members of the miR-30 family were included on the Qiagen discovery panel, but no significant variation in their expression profile was observed, including miR-30c-5p, which was used for normalisation given its consistent expression profile across all samples. Although, members of the miR-30 family share common seed motifs, variability that exists particularly at the 3´of the sequence permits targeting of different genes and pathways that influence various biological functions. The qPCR assays employed in this study were able to discriminate between miR-30 family members and miR-30c-5p was shown to be stably expressed and therefore suitable as a reference gene. Patients with renal impairment typically exhibit disturbances of lipoprotein metabolism that results in dyslipidaemia, including dysfunctional HDL-C^57^, which was positively correlated with miR-30b-5p in our data. To our knowledge, this is the first study to demonstrate an association between miR-30b-5p and impaired renal function.

Patients in the T2DKD, T2DNRF and CCKD groups were prescribed medications such as angiotensin converting enzyme (ACE) inhibitors or angiotensin II receptor blockers (ARBs), statins, oral hypoglycaemic drugs and/or insulin to manage hypertension, dyslipidaemia and diabetes respectively. There is emerging evidence that drugs influence miRNA levels *in vitro* and *in vivo*^58–60^. For instance, Macconi and colleagues demonstrated that treatment of rats with renal fibrosis using ACE inhibitors suppressed interstitial collagen deposition in the kidneys and reduced miR-324-3p expression in the tubular epithelia^61^. However, to date, there is limited data available discussing the effect of medication on miRNA expression in T2DKD.

Our data suggests that the expression profiles of miR-21-5p and miR-30b-5p are altered as a consequence of renal dysfunction and not specifically associated with DKD. The current findings indicate that increased urinary exosomal expression of miR-21-5p and decreased miR-30b-5p are collectively better able to differentiate individuals with impaired renal function. Urinary miRNAs may prove useful in the detection of early kidney damage and future monitoring of response to treatment. Earlier and longitudinal evaluation of at risk individuals may better inform the utility of these markers before the manifestation of traditional symptoms.

## Limitations

This study investigated variation of miRNA expression in individuals with T2DKD, T2DM and CKD. Classification of T2DKD was not defined by kidney biopsy and as such, associations with DKD could not be confirmed histologically. In addition, the cross-sectional nature of the study lacks an ability to examine causality and changes in miRNA profiles over time as disease progresses or the function of the candidate miRNAs identified. Further prospective evaluation of miRNA profiles, using emerging miRNA sequencing technologies, could determine how miRNAs change over time and in response to different treatment regimens as chronic kidney disease progresses.

## Summary

A global profiling approach identified altered expression of urinary exosomal miRNAs miR-21-5p and miR-30b-5p in association with poor renal function. These changes were subsequently validated in a larger independent cohort. Urinary exosomal miR-21-5p was found to be enriched in T2DKD and CKD patients compared with T2DM individuals with good renal function; while in contrast, the expression of miR-30b-5p was reduced in T2DKD and CKD patients. Both miRNAs were significantly correlated with serum creatinine levels. Urinary exosomal miR-21-5p and miR-30b-5p may represent candidate biomarkers of renal function, although further clarification is necessary to determine the extent of this association more generally across individuals with other renal conditions.

## Supplementary information

Supplementary Table S1
