# Supplementary material for: Differential Expression of Urinary Exosomal MicroRNAs miR-21-5p and miR-30b-5p in Individuals with Diabetic Kidney Disease
Source: Sci Rep. 2019 Jul 29;9:10900. doi: 10.1038/s41598-019-47504-x (PMC6662907; doi:10.1038/s41598-019-47504-x)
Supplement: Supplementary file 1 — Supplementary Table S1 [file 41598_2019_47504_MOESM1_ESM.pdf]

Differential Expression of Urinary Exosomal MicroRNAs miR-21-5p and miR-30b-5p in Individuals with Diabetic Kidney Disease

Jinnan Zang[1], Alexander P Maxwell[1], David A Simpson[2†], Gareth J McKay[1*†]

[1] *Centre for Public Health, Queen's University Belfast, Belfast, United Kingdom.*

[2] *Centre for Experimental Medicine, Queen's University Belfast, Belfast, United Kingdom.*

[†]Both authors contributed equally to this work.

*Corresponding author: g.j.mckay@qub.ac.uk

Category	Term	Count	%	P Value	Genes	Fold Enrichment	Benjamini
UP_KEYWORDS	Phosphoprotein	65	0.38	2.14E-07	CADM1, CADM2, CPEB3, EDNRB, FBXO28, RI	1.655	3.87E-05
GOTERM_BP_DIRECT	GO:1900153~positive regulation of nuclear-transcribed m	4	0.02	5.02E-05	ZFP36L2, CPEB3, AGO2, TNRC6B	53.308	0.040
UP_SEQ_FEATURE	compositionally biased region:Poly-Thr	5	0.03	9.97E-05	TRIM33, MAP3K1, BMPR2, NFAT5, PDE3A	20.472	0.039
GAD_DISEASE_CLASS	METABOLIC	44	0.26	3.33E-04	RALGPS2, LMBR1, CADM1, TSPAN2, CADM2,	1.527	0.006
GOTERM_MF_DIRECT	GO:0005515~protein binding	62	0.37	4.46E-04	CADM1, TSPAN2, CPEB3, CNOT6, EDNRB, W	1.354	0.094
UP_SEQ_FEATURE	splice variant	55	0.32	4.98E-04	CADM1, CADM2, CPEB3, EDNRB, CHIC1, RA	1.451	0.094
GOTERM_BP_DIRECT	GO:0035194~posttranscriptional gene silencing by RNA	3	0.02	5.73E-04	AGO2, AGO4, TNRC6B	79.962	0.206
UP_KEYWORDS	Bromodomain	4	0.02	8.20E-04	BRWD3, BRWD1, TRIM33, PBRM1	21.540	0.072
UP_SEQ_FEATURE	compositionally biased region:Pro-rich	14	0.08	8.46E-04	CPEB3, UBN2, GID4, MIA3, CHD7, BNC2, FOX	2.931	0.105
GAD_DISEASE	Type 2 Diabetes| edema | rosiglitazone	24	0.14	8.49E-04	RECK, CADM1, FTO, SOCS6, DAG1, SKP2, PD	1.995	0.256
UP_KEYWORDS	Chromosomal rearrangement	8	0.05	0.001	BRWD3, SATB1, ERG, TRIM33, BCL2, LIFR, H	5.030	0.060
GOTERM_BP_DIRECT	GO:0007223~Wnt signaling pathway, calcium modulating	4	0.02	0.001	AGO2, AGO4, PPP3CA, TNRC6B	19.136	0.265
GAD_DISEASE_CLASS	PHARMACOGENOMIC	27	0.16	0.001	CADM1, DAG1, RFFL, NHS, TIMP3, EDNRB, B	1.815	0.011
INTERPRO	IPR001487:Bromodomain	4	0.02	0.001	BRWD3, BRWD1, TRIM33, PBRM1	18.173	0.259
INTERPRO	IPR007527:Zinc finger, SWIM-type	3	0.02	0.001	BNC2, MAP3K1, ZSWIM6	53.279	0.140
UP_SEQ_FEATURE	domain:Bromo 1	3	0.02	0.001	BRWD3, BRWD1, PBRM1	51.181	0.136
UP_SEQ_FEATURE	domain:Bromo 2	3	0.02	0.001	BRWD3, BRWD1, PBRM1	51.181	0.136
GOTERM_BP_DIRECT	GO:0060213~positive regulation of nuclear-transcribed m	3	0.02	0.001	CPEB3, AGO2, TNRC6B	50.885	0.258
UP_KEYWORDS	Translation regulation	5	0.03	0.002	CPEB3, AGO2, AGO4, TNRC6B, CNOT6	10.000	0.067
GOTERM_BP_DIRECT	GO:0030336~negative regulation of cell migration	5	0.03	0.002	RECK, MIA3, BCL2, DAG1, RHOB	9.820	0.230
GOTERM_BP_DIRECT	GO:0035278~miRNA mediated inhibition of translation	3	0.02	0.002	AGO2, AGO4, TNRC6B	46.644	0.212
UP_KEYWORDS	RNA-mediated gene silencing	4	0.02	0.002	AGO2, AGO4, TNRC6B, CNOT6	15.850	0.070
UP_KEYWORDS	Nucleus	39	0.23	0.002	CPEB3, DAG1, CNOT6, ZFP36L2, CHD7, RAVE	1.562	0.060

SMART	SM00297:BROMO	4	0.02	0.002	BRWD3, BRWD1, TRIM33, PBRM1	15.203	0.146
UP_KEYWORDS	Lipoprotein	12	0.07	0.002	RECK, EDNRB, CHIC1, RAB22A, LRP6, RHOB,	2.958	0.056
UP_KEYWORDS	Alternative splicing	65	0.38	0.003	CADM1, TSPAN2, CADM2, CPEB3, EDNRB, Cl	1.289	0.057
UP_KEYWORDS	Zinc	22	0.13	0.003	KLF12, KLF9, TRPM7, ZSWIM6, MBNL1, RFFL	1.968	0.053
UP_SEQ_FEATURE	compositionally biased region:Poly-Ser	9	0.05	0.003	BRWD1, MAP3K1, BMPR2, SOCS6, ZSWIM6, T	3.620	0.227
GOTERM_CC_DIRECT	GO:0005829~cytosol	29	0.17	0.004	DAG1, RFFL, FAM13A, CNOT6, ZFP36L2, UBE	1.678	0.451
GOTERM_BP_DIRECT	GO:0045944~positive regulation of transcription from RN	13	0.08	0.005	KLF12, BMPR2, TET1, CHD7, BCL11B, NFAT5	2.472	0.469
GOTERM_CC_DIRECT	GO:0031965~nuclear membrane	6	0.04	0.007	EDNRB, BCL2, TNKS, TNPO1, AKIRIN1, TNPC	5.026	0.384
UP_KEYWORDS	Repressor	9	0.05	0.007	SATB1, TRIM33, KLF12, CPEB3, BCL11B, AGC	3.193	0.117
UP_SEQ_FEATURE	compositionally biased region:Poly-Gln	5	0.03	0.007	SATB1, ZFP36L2, BRWD1, NFAT5, GLCCI1	6.562	0.368
UP_KEYWORDS	Protein transport	9	0.05	0.008	MIA3, AP3M1, RAB22A, RHOB, RAB11A, TNK	3.099	0.126
UP_KEYWORDS	Metal-binding	28	0.17	0.009	BMPR2, RFFL, TIMP3, CNOT6, ZFP36L2, ZNF7	1.615	0.124
GOTERM_BP_DIRECT	GO:0033077~T cell differentiation in thymus	3	0.02	0.009	ZFP36L2, BCL11B, BCL2	20.731	0.595
GOTERM_CC_DIRECT	GO:0005634~nucleus	40	0.24	0.010	CPEB3, TIMP3, CNOT6, ZFP36L2, ZNF704, CH	1.417	0.378
UP_SEQ_FEATURE	zinc finger region:C3H1-type 1	3	0.02	0.010	ZFP36L2, MBNL1, MBNL3	19.812	0.426
UP_SEQ_FEATURE	zinc finger region:C3H1-type 2	3	0.02	0.010	ZFP36L2, MBNL1, MBNL3	19.812	0.426
UP_KEYWORDS	Tumor suppressor	5	0.03	0.010	RECK, CADM1, RHOB, PBRM1, RASA1	5.932	0.129
GOTERM_BP_DIRECT	GO:0016340~calcium-dependent cell-matrix adhesion	2	0.01	0.011	TRPM7, DAG1	186.578	0.614
GOTERM_BP_DIRECT	GO:1904684~negative regulation of metalloendopeptidase	2	0.01	0.011	RECK, TIMP3	186.578	0.614
GOTERM_BP_DIRECT	GO:0006338~chromatin remodeling	4	0.02	0.011	SATB1, CHD7, NUDT5, PBRM1	8.678	0.580
UP_KEYWORDS	Chromatin regulator	6	0.04	0.012	SATB1, CHD7, PBRM1, HDAC9, TET1, BAHD1	4.390	0.140
UP_KEYWORDS	RNA-binding	9	0.05	0.013	ZFP36L2, CPEB3, RAVER2, NUDT5, AGO2, AC	2.842	0.149
GAD_DISEASE	Tobacco Use Disorder	26	0.15	0.013	LMBR1, TSPAN2, CADM1, CADM2, CPEB3, BI	1.566	0.903
UP_KEYWORDS	Ubl conjugation	16	0.09	0.013	SATB1, PELI1, CPEB3, SKP2, WNK3, RFFL, TF	1.971	0.142
UP_SEQ_FEATURE	compositionally biased region:Poly-Ala	7	0.04	0.014	ZFP36L2, CHD7, MAP3K1, SNTB2, PDE3A, GL	3.547	0.492
GOTERM_CC_DIRECT	GO:0045202~synapse	5	0.03	0.014	CADM1, CPEB3, CADM2, LRP6, SNTB2	5.299	0.408
GAD_DISEASE	Body Mass Index	8	0.05	0.014	TSPAN2, CPEB3, CADM2, FTO, TRMT5, FAM4	3.051	0.816
UP_KEYWORDS	Ubl conjugation pathway	9	0.05	0.015	PELI1, UBE2D3, TRIM33, WWP1, FBXO28, SO	2.780	0.149
GOTERM_MF_DIRECT	GO:0003682~chromatin binding	7	0.04	0.016	SATB1, ERG, CHD7, PYGO1, PBRM1, NFIA, BA	3.434	0.823
GAD_DISEASE_CLASS	CARDIOVASCULAR	33	0.19	0.017	CADM1, CADM2, BMPR2, RFFL, FAM46A, FA	1.413	0.092
GAD_DISEASE	Echocardiography	7	0.04	0.018	CHD7, BCL2, RHOB, SEMA3A, TNRC6B, NEGI	3.313	0.788
UP_KEYWORDS	Palmitate	6	0.04	0.019	EDNRB, CHIC1, LRP6, RHOB, RFFL, PAG1	3.889	0.172
GOTERM_BP_DIRECT	GO:0048015~phosphatidylinositol-mediated signaling	4	0.02	0.019	AGO2, AGO4, TNRC6B, FRS2	7.041	0.750
UP_SEQ_FEATURE	DNA-binding region:CTF/NF-I	2	0.01	0.019	NFIA, NFIB	102.362	0.572

UP_KEYWORDS	Zinc-finger	16	0.09	0.019	KLF9, KLF12, ZSWIM6, MBNL1, RFFL, TET1, Z	1.887	0.170
INTERPRO	IPR000647:CTF transcription factor/nuclear factor 1	2	0.01	0.020	NFIA, NFIB	97.679	0.779
INTERPRO	IPR019548:CTF transcription factor/nuclear factor 1, N-te	2	0.01	0.020	NFIA, NFIB	97.679	0.779
INTERPRO	IPR020604:CTF transcription factor/nuclear factor 1, DNA	2	0.01	0.020	NFIA, NFIB	97.679	0.779
INTERPRO	IPR019739:CTF transcription factor/nuclear factor 1, cons	2	0.01	0.020	NFIA, NFIB	97.679	0.779
UP_KEYWORDS	Cell membrane	24	0.14	0.021	RECK, RALGPS2, CADM1, CADM2, CPEB3, BN	1.587	0.175
GOTERM_BP_DIRECT	GO:0090625~mRNA cleavage involved in gene silencing	2	0.01	0.021	AGO2, AGO4	93.289	0.760
GOTERM_BP_DIRECT	GO:0016569~covalent chromatin modification	4	0.02	0.022	CHD7, PBRM1, TET1, BAHD1	6.605	0.751
GAD_DISEASE	Body Weight	7	0.04	0.023	CHD7, FTO, HDAC9, NHS, NEGR1, NFIA, NFIB	3.119	0.802
UP_KEYWORDS	Transcription regulation	19	0.11	0.024	SATB1, ERG, KLF12, KLF9, TET1, CNOT6, BRV	1.711	0.187
GOTERM_BP_DIRECT	GO:0070936~protein K48-linked ubiquitination	3	0.02	0.026	PELI1, UBE2D3, RFFL	11.909	0.777
GOTERM_BP_DIRECT	GO:0035279~mRNA cleavage involved in gene silencing	2	0.01	0.026	AGO2, AGO4	74.631	0.760
KEGG_PATHWAY	hsa04120:Ubiquitin mediated proteolysis	4	0.02	0.029	UBE2D3, WWP1, MAP3K1, SKP2	5.738	0.911
GOTERM_BP_DIRECT	GO:0006357~regulation of transcription from RNA polym	7	0.04	0.030	BRWD3, ERG, BRWD1, ZNF704, KLF12, KLF9,	2.962	0.780
INTERPRO	IPR014811:Domain of unknown function DUF1785	2	0.01	0.030	AGO2, AGO4	65.119	0.817
UP_KEYWORDS	Transcription	19	0.11	0.030	SATB1, ERG, KLF12, KLF9, TET1, CNOT6, BRV	1.664	0.224
SMART	SM01163:SM01163	2	0.01	0.030	AGO2, AGO4	63.854	0.677
GOTERM_CC_DIRECT	GO:0035068~micro-ribonucleoprotein complex	2	0.01	0.031	AGO2, AGO4	63.944	0.593
GAD_DISEASE_CLASS	VISION	9	0.05	0.032	EDNRB, KLF12, FTO, LRP6, PPP3CA, SEMA3A	2.386	0.127
INTERPRO	IPR001806:Small GTPase superfamily	4	0.02	0.034	RASEF, RAB22A, RHOB, RAB11A	5.622	0.783
GOTERM_MF_DIRECT	GO:0043734~DNA-N1-methyladenine dioxygenase activi	2	0.01	0.036	FTO, TET1	54.808	0.930
GOTERM_CC_DIRECT	GO:0070578~RISC-loading complex	2	0.01	0.036	AGO2, AGO4	54.809	0.583
INTERPRO	IPR000571:Zinc finger, CCCH-type	3	0.02	0.036	ZFP36L2, MBNL1, MBNL3	9.933	0.744
GAD_DISEASE_CLASS	CHEMDEPENDENCY	28	0.17	0.036	LMBR1, TSPAN2, CADM1, CADM2, CPEB3, BN	1.406	0.119
GOTERM_BP_DIRECT	GO:0071679~commissural neuron axon guidance	2	0.01	0.037	DAG1, NFIB	53.308	0.828
GOTERM_BP_DIRECT	GO:0050798~activated T cell proliferation	2	0.01	0.037	SATB1, CADM1	53.308	0.828
GOTERM_BP_DIRECT	GO:0010586~miRNA metabolic process	2	0.01	0.037	AGO2, AGO4	53.308	0.828
GOTERM_BP_DIRECT	GO:0035280~miRNA loading onto RISC involved in gene	2	0.01	0.037	AGO2, AGO4	53.308	0.828
SMART	SM00356:ZnF_C3H1	3	0.02	0.037	ZFP36L2, MBNL1, MBNL3	9.774	0.596
GOTERM_BP_DIRECT	GO:0000122~negative regulation of transcription from RN	9	0.05	0.037	EDNRB, SATB1, UBE2D3, TRIM33, KLF12, CP	2.332	0.814
GOTERM_MF_DIRECT	GO:0003725~double-stranded RNA binding	3	0.02	0.039	AGO2, AGO4, MBNL1	9.434	0.892
INTERPRO	IPR003165:Stem cell self-renewal protein Piwi	2	0.01	0.040	AGO2, AGO4	48.839	0.726
GOTERM_BP_DIRECT	GO:0019934~cGMP-mediated signaling	2	0.01	0.042	EDNRB, PDE3A	46.644	0.835
GOTERM_BP_DIRECT	GO:0035196~production of miRNAs involved in gene sile	2	0.01	0.042	AGO2, AGO4	46.644	0.835

GAD_DISEASE	systemic lupus erythematosus	4	0.02	0.042	BCL2, SOCS6, NEGR1, TNPO3	5.110	0.917
UP_SEQ_FEATURE	zinc finger region:SWIM-type	2	0.01	0.043	MAP3K1, ZSWIM6	45.494	0.821
UP_SEQ_FEATURE	domain:Piwi	2	0.01	0.043	AGO2, AGO4	45.494	0.821
GAD_DISEASE_CLASS	CANCER	25	0.15	0.045	CADM2, BMPR2, FAM46A, TIMP3, EDNRB, M	1.429	0.121
GAD_DISEASE	Respiratory Function Tests	5	0.03	0.045	CADM1, TRPM7, CADM2, PBRM1, FAM13A	3.683	0.898
UP_KEYWORDS	Methylation	10	0.06	0.046	MIA3, CHD7, TRIM33, KLF12, CPEB3, BCL11E	2.098	0.312
UP_SEQ_FEATURE	domain:PAZ	2	0.01	0.047	AGO2, AGO4	40.945	0.824
GAD_DISEASE	Sclerosis	2	0.01	0.048	RHOB, TNPO3	40.598	0.880
SMART	SM00950:SM00950	2	0.01	0.048	AGO2, AGO4	39.909	0.595
GAD_DISEASE_CLASS	NEUROLOGICAL	23	0.14	0.048	RECK, SNX29, CADM1, CADM2, CPEB3, TRPM	1.454	0.114
GAD_DISEASE	Osteoarthritis	3	0.02	0.049	KLF12, LRP6, RHOB	8.304	0.859
INTERPRO	IPR003100:Argonaute/Dicer protein, PAZ	2	0.01	0.050	AGO2, AGO4	39.072	0.757
GOTERM_MF_DIRECT	GO:0005068~transmembrane receptor protein tyrosine kir	2	0.01	0.050	FRS2, PAG1	38.366	0.898
GOTERM_BP_DIRECT	GO:0006351~transcription, DNA-templated	17	0.10	0.050	SATB1, ERG, KLF12, KLF9, TET1, CNOT6, BR	1.622	0.876
UP_KEYWORDS	Disease mutation	19	0.11	0.051	FTO, BMPR2, ZSWIM6, DAG1, LIFR, PDE3A, M	1.565	0.326
BIOCARTA	h_dicerPathway:Dicer Pathway	2	0.01	0.051	AGO2, AGO4	36.111	0.972
GOTERM_BP_DIRECT	GO:0003197~endocardial cushion development	2	0.01	0.052	ERG, BMPR2	37.316	0.870
INTERPRO	IPR005225:Small GTP-binding protein domain	4	0.02	0.053	RASEF, RAB22A, RHOB, RAB11A	4.679	0.740
UP_SEQ_FEATURE	compositionally biased region:Poly-Gly	5	0.03	0.053	ZFP36L2, TRIM33, MAP3K1, GLCCI1, RASA1	3.506	0.834
UP_KEYWORDS	Wnt signaling pathway	4	0.02	0.054	PYGO1, LRP6, ROR1, TNKS	4.641	0.332
SMART	SM00949:SM00949	2	0.01	0.054	AGO2, AGO4	35.474	0.556
INTERPRO	IPR001025:Bromo adjacent homology (BAH) domain	2	0.01	0.054	PBRM1, BAHD1	35.520	0.712
GOTERM_MF_DIRECT	GO:0035925~mRNA 3'-UTR AU-rich region binding	2	0.01	0.055	ZFP36L2, CPEB3	34.878	0.877
GOTERM_CC_DIRECT	GO:0016442~RISC complex	2	0.01	0.055	AGO2, AGO4	34.878	0.692
UP_SEQ_FEATURE	domain:MHD	2	0.01	0.057	AP3M1, AP5M1	34.121	0.828
GOTERM_BP_DIRECT	GO:0050868~negative regulation of T cell activation	2	0.01	0.057	SOCS6, PAG1	33.923	0.882
GOTERM_BP_DIRECT	GO:0009791~post-embryonic development	3	0.02	0.057	BCL2, PYGO1, AGO2	7.668	0.873
UP_KEYWORDS	Transferase	14	0.08	0.057	TRPM7, NUDT5, BMPR2, SKP2, WNK3, SAMD	1.721	0.338
INTERPRO	IPR003619:MAD homology 1, Dwarfin-type	2	0.01	0.059	NFIA, NFIB	32.560	0.709
GOTERM_CC_DIRECT	GO:0000932~cytoplasmic mRNA processing body	3	0.02	0.061	AGO2, AGO4, TNRC6B	7.378	0.683
GOTERM_BP_DIRECT	GO:0060021~palate development	3	0.02	0.062	CHD7, BNC2, LRP6	7.365	0.881
GOTERM_BP_DIRECT	GO:0061158~3'-UTR-mediated mRNA destabilization	2	0.01	0.062	ZFP36L2, CPEB3	31.096	0.872
GOTERM_BP_DIRECT	GO:0070989~oxidative demethylation	2	0.01	0.062	FTO, TET1	31.096	0.872
GOTERM_BP_DIRECT	GO:0050678~regulation of epithelial cell proliferation	2	0.01	0.062	EDNRB, FRS2	31.096	0.872

GOTERM_MF_DIRECT	GO:0046872~metal ion binding	17	0.10	0.062	KLF9, KLF12, TRPM7, BMPR2, PDE3A, MBNL	1.576	0.868
UP_SEQ_FEATURE	zinc finger region:C3H1-type 4	2	0.01	0.066	MBNL1, MBNL3	29.246	0.852
SMART	SM00439:BAH	2	0.01	0.066	PBRM1, BAHD1	29.025	0.563
GOTERM_BP_DIRECT	GO:0031054~pre-miRNA processing	2	0.01	0.067	AGO2, AGO4	28.704	0.882
INTERPRO	IPR023152:Ras GTPase-activating protein, conserved site	2	0.01	0.069	RASA1, RASA2	27.908	0.733
INTERPRO	IPR003585:Neurexin/syndecan/glycophorin C	2	0.01	0.069	CADM1, CADM2	27.908	0.733
GOTERM_CC_DIRECT	GO:0005737~cytoplasm	35	0.21	0.069	RALGPS2, CPEB3, BMPR2, DAG1, RFFL, NHS,	1.286	0.683
UP_SEQ_FEATURE	domain:Ras-GAP	2	0.01	0.070	RASA1, RASA2	27.297	0.852
GOTERM_CC_DIRECT	GO:0010008~endosome membrane	4	0.02	0.071	UBE2D3, RAB22A, RHOB, RFFL	4.148	0.654
SMART	SM00523:DWA	2	0.01	0.072	NFIA, NFIB	26.606	0.539
GOTERM_MF_DIRECT	GO:0061630~ubiquitin protein ligase activity	4	0.02	0.072	PELI1, WWP1, SKP2, RFFL	4.103	0.874
GOTERM_CC_DIRECT	GO:0005654~nucleoplasm	21	0.12	0.074	SATB1, KLF12, KLF9, DAG1, SKP2, MBNL1, R	1.447	0.638
GOTERM_BP_DIRECT	GO:0000209~protein polyubiquitination	4	0.02	0.074	UBE2D3, BCL2, SKP2, TNKS	4.056	0.900
GOTERM_CC_DIRECT	GO:0031235~intrinsic component of the cytoplasmic side	2	0.01	0.075	RASA1, RASA2	25.578	0.608
BIOCARTA	h_tcrPathway:T Cell Receptor Signaling Pathway	3	0.02	0.075	MAP3K1, PPP3CA, RASA1	6.155	0.930
UP_KEYWORDS	Endosome	6	0.04	0.077	UBE2D3, RAB22A, RHOB, RAB11A, RFFL, AP5	2.620	0.416
INTERPRO	IPR008936:Rho GTPase activation protein	3	0.02	0.078	FAM13A, RASA1, RASA2	6.440	0.751
INTERPRO	IPR001936:Ras GTPase-activating protein	2	0.01	0.078	RASA1, RASA2	24.420	0.726
GOTERM_CC_DIRECT	GO:0001726~ruffle	3	0.02	0.079	TRPM7, RAB22A, RASA1	6.394	0.599
GOTERM_MF_DIRECT	GO:0035198~miRNA binding	2	0.01	0.079	AGO2, AGO4	23.979	0.869
GOTERM_MF_DIRECT	GO:0008191~metalloendopeptidase inhibitor activity	2	0.01	0.079	RECK, TIMP3	23.979	0.869
GOTERM_CC_DIRECT	GO:0016010~dystrophin-associated glycoprotein complex	2	0.01	0.079	DAG1, SNTB2	23.979	0.576
GOTERM_CC_DIRECT	GO:0030014~CCR4-NOT complex	2	0.01	0.079	CPEB3, CNOT6	23.979	0.576
GOTERM_BP_DIRECT	GO:0007420~brain development	4	0.02	0.080	TSPAN2, CADM1, FOXG1, BMPR2	3.928	0.909
GOTERM_BP_DIRECT	GO:0006915~apoptotic process	7	0.04	0.080	UBE2D3, CADM1, BCL2, MAP3K1, RHOB, RFF	2.303	0.902
GOTERM_BP_DIRECT	GO:0080111~DNA demethylation	2	0.01	0.082	FTO, TET1	23.322	0.898
UP_KEYWORDS	Kallmann syndrome	2	0.01	0.082	CHD7, SEMA3A	23.334	0.423
SMART	SM00323:RasGAP	2	0.01	0.083	RASA1, RASA2	22.805	0.546
SMART	SM00294:4.1m	2	0.01	0.083	CADM1, CADM2	22.805	0.546
UP_KEYWORDS	Cytoplasm	30	0.18	0.084	RALGPS2, CPEB3, DAG1, RFFL, NHS, CNOT6,	1.308	0.422
UP_SEQ_FEATURE	short sequence motif:Effector region	3	0.02	0.086	RAB22A, RHOB, RAB11A	6.081	0.891
GOTERM_BP_DIRECT	GO:0071310~cellular response to organic substance	2	0.01	0.086	BCL2, TIMP3	21.950	0.904
BIOCARTA	h_reckPathway:Inhibition of Matrix Metalloproteinases	2	0.01	0.091	RECK, TIMP3	20.062	0.884
GOTERM_BP_DIRECT	GO:0006402~mRNA catabolic process	2	0.01	0.091	ZFP36L2, AGO4	20.731	0.910

UP_KEYWORDS	Activator	7	0.04	0.092	BRWD1, CPEB3, NFAT5, CNOT6, TET1, NFIA,	2.224	0.442
GOTERM_BP_DIRECT	GO:0043066~negative regulation of apoptotic process	6	0.04	0.094	EDNRB, BCL11B, BCL2, WNK3, PDE3A, AGO4	2.460	0.910
GOTERM_MF_DIRECT	GO:0002020~protease binding	3	0.02	0.096	BCL2, RFFL, TIMP3	5.698	0.892
GOTERM_BP_DIRECT	GO:0021772~olfactory bulb development	2	0.01	0.096	CHD7, SEMA3A	19.640	0.909
UP_KEYWORDS	Membrane	43	0.25	0.097	RALGPS2, LMBR1, CADM1, TSPAN2, CADM2,	1.205	0.447
UP_SEQ_FEATURE	zinc finger region:C3H1-type 3	2	0.01	0.097	MBNL1, MBNL3	19.498	0.906
GAD_DISEASE	weight	2	0.01	0.098	FTO, NEGR1	19.231	0.972
